# Influenza vaccination reduces incidence of peripheral arterial occlusive disease in elderly patients with chronic kidney disease

**DOI:** 10.1038/s41598-021-84285-8

**Published:** 2021-03-01

**Authors:** Ping-Jen Hu, Chia-Hsien Chen, Chung-Shun Wong, Tzu-Ting Chen, Mei-Yi Wu, Li-Chin Sung

**Affiliations:** 1 Division of Gastroenterology and Hepatology, Department of Internal Medicine, Taitung Mackay Memorial Hospital, Taitung, Taiwan; 2 Division of Gastroenterology, Department of Internal Medicine, Shuang Ho Hospital, Taipei Medical University, New Taipei City, Taiwan; 3 Master Program in Biomedicine, College of Science and Engineering, National Taitung University, Taitung, Taiwan; 4 Department of Orthopedics, Shuang Ho Hospital, Taipei Medical University, New Taipei, Taiwan; 5 Department of Orthopedic Surgery, School of Medicine, College of Medicine, Taipei Medical University, Taipei, Taiwan; 6 School of Biomedical Engineering, College of Biomedical Engineering, Taipei Medical University, Taipei, Taiwan; 7 Graduate Institute of Clinical Medicine, College of Medicine, Taipei Medical University, Taipei, Taiwan; 8 Emergency Department, Shuang Ho Hospital, Taipei Medical University, New Taipei City, Taiwan; 9 Department of Emergency, School of Medicine, College of Medicine, Taipei Medical University, Taipei, Taiwan; 10 Institute of Epidemiology and Preventive Medicine, College of Public Health, National Taiwan University, Taipei, Taiwan; 11 Center for Neuropsychiatric Research, National Health Research Institutes, Miaoli, Taiwan; 12 Department of Primary Care Medicine, Shuang Ho Hospital, Taipei Medical University, New Taipei City, Taiwan; 13 Division of Nephrology, Department of Internal Medicine, School of Medicine, College of Medicine, Taipei Medical University, Taipei, Taiwan; 14 Division of Nephrology, Department of Internal Medicine, Shuang Ho Hospital, Taipei Medical University, New Taipei City, Taiwan; 15 TMU Research Center of Urology and Kidney, Taipei Medical University, Taipei, Taiwan; 16 Division of Cardiology, Department of Internal Medicine, School of Medicine, College of Medicine, Taipei Medical University, Taipei, Taiwan; 17 Division of Cardiology, Department of Internal Medicine, Shuang Ho Hospital, Taipei Medical University, New Taipei City, Taiwan; 18 Taipei Heart Institute, Taipei Medical University, Taipei, Taiwan

**Keywords:** Vaccines, Peripheral vascular disease, Chronic kidney disease

## Abstract

An influenza vaccination might reduce the risk of incident peripheral arterial occlusive disease (PAOD) in patients with chronic kidney disease (CKD), but supporting evidence is limited. This case-crossover study analyzed data from Taiwan’s real-world National Health Insurance Research Database. This study included elderly (≥ 67 years old) patients with CKD having incident PAOD from January 1, 2006, to June 30, 2015. We defined 1 year before PAOD onset as the index date for the self-control group. A conditional logistic regression model was used to investigate exposure to an influenza vaccination for estimating the risk for incident PAOD following vaccination. In total, this study included 46,782 elderly patients with CKD having incident PAOD. The odds ratios for incident PAOD were 0.85 (95% confidence interval 0.77–0.94), 0.85 (0.79–0.92), 0.84 (0.79–0.90), and 0.85 (0.81–0.90) at 1, 2, 3, and 4 months after an influenza vaccination, respectively. We observed consistent results for the subgroups of patients with CKD and concomitant diabetes. However, we did not observe any beneficial effects of influenza vaccination in patients with advanced CKD or end-stage renal disease. This study demonstrated that influenza vaccination may be associated with a reduced risk of incident PAOD among patients with early-stage CKD.

## Introduction

Chronic kidney disease (CKD), a chronic inflammatory disease, is a critical global public health concern because of its high risk of progression to end-stage renal disease (ESRD) and its poor morbidity and mortality^1,2^. Taiwan has the highest incidence and prevalence rates of CKD and ESRD worldwide^2,3^. Additionally, diabetes mellitus (DM) is the primary cause of ESRD in 40–50% of patients in Taiwan^3^. Patients with CKD have a higher rate of cardiovascular mortality, which is predominantly associated with atherosclerotic cardiovascular disease (ASCVD), including coronary artery disease (CAD), stroke, and peripheral arterial occlusive disease (PAOD), than age-matched controls without CKD^4^.

Increasing evidence has revealed that CKD and ESRD are key risk factors for noncardiovascular morbidity, and after cardiovascular disease, infection is the second leading cause of hospitalization^5–7^. Patients with CKD are at a high risk of contracting influenza^6^. Among elderly individuals aged 65 years or older, those with CKD are at a higher risk of major influenza-related complications considering their altered immune response and persistent low-grade inflammation^5,8^. Major complications of influenza can lead to hospitalization or even cardiovascular mortality. Observational studies have demonstrated that influenza vaccination can reduce the risk of first acute coronary syndrome (ACS), the hospitalization rate, and the mortality risk among elderly patients with CKD or ESRD^8^. In clinical practice guidelines, annual influenza vaccination is recommended for all adults with CKD^5^.

PAOD is a systemic atherosclerotic process, with the same critical risk factors as those for CAD and stroke. Major risk factors for PAOD include DM, hypertension, dyslipidemia, advanced age, and cigarette smoking^9^. PAOD prevalence increases with age; it is estimated to be 10% in adults older than 55 years^10^. PAOD comprises a wide range of clinical presentations from asymptomatic lesions to critical limb ischemia. However, patients with PAOD are usually asymptomatic and their conditions may remain undiagnosed. The majority of patients with PAOD also develop severe CAD^11^. Studies have demonstrated that influenza vaccination can reduce the risk of recurrent major cardiovascular events in patients with CAD^12,13^. On the basis of this evidence, annual influenza vaccination is recommended as a class 1 indication for patients with PAOD, despite the lack of direct evidence^14^.

Recent studies have revealed an increased risk of PAOD in patients with CKD^15,16^. The protective effect of a vaccine for preventing PAOD progression in elderly patients with CKD is not completely understood. To assess the potential benefit of influenza vaccination for reducing PAOD risk in elderly patients with CKD, we conducted a population-based case-crossover study by using reimbursement claims data from the National Health Insurance (NHI) Research Database (NHIRD) in Taiwan.

## Materials and methods

### Data source

In this case-crossover study, data were obtained from the NHIRD in Taiwan between January 2004 and December 2015. Taiwan’s NHI program was implemented in March 1995 and covered 99.6% of the Taiwanese population of 23 million by 2015. In the NHIRD, the data of registration files and medical claims for all beneficiaries can be linked through encrypted identification numbers (details available at http://nhird.nhri.org.tw/en/index.htm). Diseases in the NHIRD were identified using International Classification of Diseases, Ninth Revision, Clinical Modification (ICD-9-CM) codes (until 2015). Detailed description and reviews of this database are provided elsewhere^17^. The current study was approved by the Taipei Medical University-Joint Institutional Review Board (TMU-JIRB No.: N201908050). The requirement for informed consent was waived by the TMU-JIRB because all data from the NHIRD have been anonymized. Our research was performed in accordance with the relevant guidelines and regulations.

### Study population

**Figure 1:**
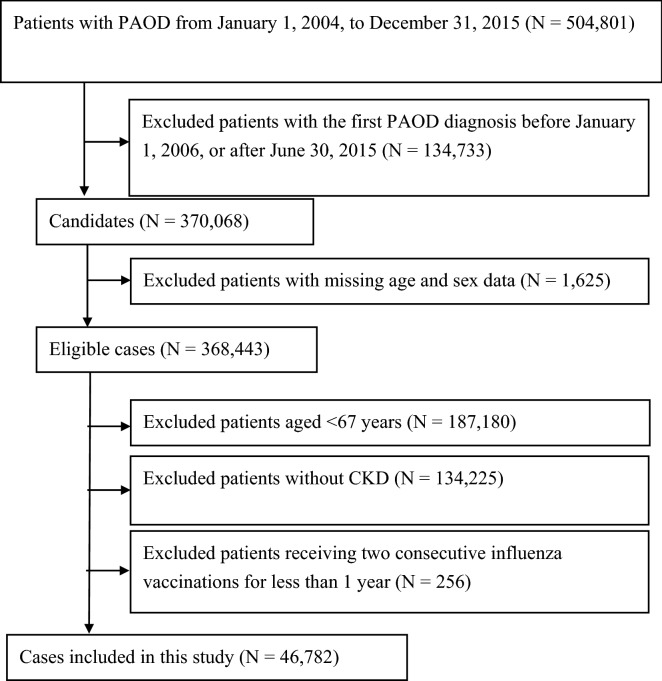
Flowchart of the selection criteria and process for eligible patients with CKD and PAOD. *CKD* chronic kidney disease, *PAOD* peripheral arterial occlusive disease.

We identified all patients with CKD having incident PAOD between January 1, 2006, and June 30, 2015. We used a 2-year washout period (2004–2005) to ensure that patients with CKD did not have a prior PAOD diagnosis (Fig. 1). Patients were considered to have PAOD if they had at least three outpatient visits or at least one hospitalization associated with the following ICD-9-CM codes: 440.2, 440.3, 440.8, 440.9, 443, 444.22, 444.8, 447.8, or 447.9. Patients with CKD, including CKD patients whose disease progressed to ESRD, were identified as those with at least three outpatient claims records or at least one inpatient claims record with the following ICD-9-CM codes before PAOD onset: 250.4, 274.1, 283.11, 403, 404, 440.1, 442.1, 447.3, 581–582, 585–589, 642.1, or 646.2. We did not include chronic pyelonephritis as CKD patients due to coexisting chronic inflammation and pyogenic infection. Besides, polycystic kidney is an inherited, multisystemic, and progressive disorder characterized by cyst formation and enlargement of the kidney and other organs (e.g., liver, pancreas, and spleen), which was also excluded in our study. Patients with ESRD were identified as those receiving dialysis without a kidney transplant before PAOD onset.

### Seasonal influenza vaccination

In Taiwan, the influenza vaccination has been free of charge and recommended for the high-risk elderly population since 1998 and for all adults older than 65 years since 2001^8^. The vaccine usually includes a mixture of two influenza A strains and one or two influenza B strains. Almost all people in Taiwan receive the same components of the seasonal influenza vaccine. The vaccination status was identified using the ICD-9-CM code V04.8 and/or the use of a vaccine (confirmed based on drug codes).

### case-crossover design and exposure measurement

The case-crossover design is a special type of case-only study, which includes only cases, and the cases serve as their own control^18,19^. Time-invariant confounders are implicitly adjusted for because of the within-person comparison. We defined the 1 year before the onset of PAOD as the index date for the self-control group. The extra 1 year period before the index day of self-control is essential for the comparison with PAOD case. For the above reasons, we recruited patients with incident PAOD when they were 67 years or older, assuring that all study population are eligible to receive free influenza vaccines. A previous study demonstrated that the effect of the vaccine does not commence within the initial 2 weeks of administration^20^. Therefore, we did not consider 2 weeks prior to the incident PAOD diagnosis and index date as the exposure window (Supplementary Fig. S1). The effect of influenza vaccination was measured from 2 weeks before the index date to 6 weeks before the index date for a 1-month exposure window in both the case and control groups. We considered the length of the exposure window to be either 1, 2, 3, or 4 months prior to 2 weeks before the index date for both the case and control groups. We also excluded patients who received two consecutive influenza vaccinations within less than 1 year due to the possible misclassification of no exposure in the self-control group in the case-crossover design (Supplementary Fig. S1). Moreover, patients with missing data for age or sex, those aged less than 67 years, or those without CKD were excluded (Fig. 1).

### Covariates

We identified DM based on the presence of at least 1 admission record or at least 3 outpatient visits with the ICD-9-CM code 250 within 1 year before the index date in both the case and control groups. In this study, advanced CKD was defined as the presence of the relevant codes for CKD and concomitant reimbursements for prescriptions of erythropoiesis-stimulating agents (ESAs), which are initiated when patients with CKD have serum creatinine levels of > 6 mg/dL (approximately equivalent to the glomerular filtration rate [GFR] of < 15 mL/min per 1.73 m^2^) and hematocrit of < 28%^21^; in the absence of treatment with an ESA, early-stage CKD was confirmed^21^.

### Statistical analysis

We first estimated the association between influenza vaccination and the risk of incident PAOD by using a conditional logistic regression model and subsequently stratified the study population by sex. We also performed subgroup analyses among patients with DM, early-stage CKD, or advanced CKD/ESRD. To reduce selection bias, we excluded patients who were hospitalized for more than 30 days within 1 year prior to PAOD onset, because influenza vaccination may be delayed or may not even be provided to these patients due to their fragile status. All analyses were performed using the SAS statistical software package (SAS System for Windows, Version 9.4, SAS Institute Inc., Cary, NC, USA).

## Results

**Figure 2:**
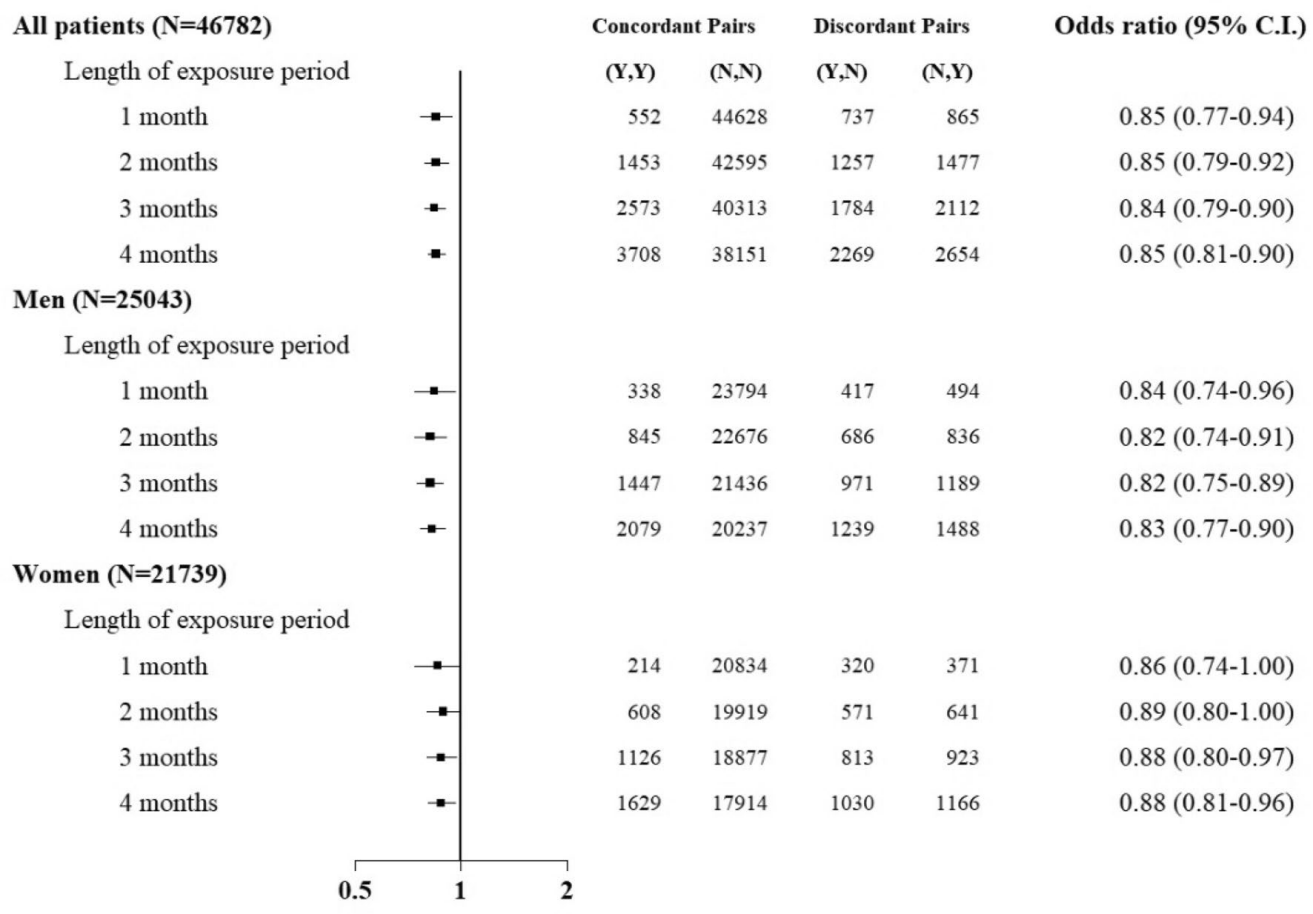
ORs for incident PAOD after influenza vaccination in patients with CKD when the control was selected from 1 year before PAOD. *CKD* chronic kidney disease, *PAOD* peripheral arterial occlusive disease, *ORs* odds ratios.

After excluding patients with missing data, we identified 46,782 elderly patients with CKD having incident PAOD. The flowchart is shown in Fig. 1. The characteristics of these patients are shown in Table 1. The mean age of patients with CKD having incident PAOD was 77.6 ± 6.7 years. Figure 2 shows that the odds ratios (ORs) and 95% confidence intervals (CIs; in parentheses) for PAOD in the study population were 0.85 (0.77–0.94), 0.85 (0.79–0.92), 0.84 (0.79–0.90), and 0.85 (0.81–0.90) for the exposure windows of 1, 2, 3, and 4 months, respectively. For men, the ORs were 0.84 0.74–0.96), 0.82 (0.74–0.91), 0.82 (0.75–0.89), and 0.83 (0.77–0.90) for the exposure windows of 1, 2, 3, and 4 months, respectively. For women, the ORs were 0.86 (0.74–1.00), 0.89 (0.80–1.00), 0.88 (0.80–0.97), and 0.88 (0.81–0.96) for the exposure windows of 1, 2, 3, and 4 months, respectively.

**Table 1:** Characteristics of patients with PAOD.

Characteristics	Case		Self-control
N	46,782		
Age at first PAOD, mean ± SD, y	77.6 ± 6.7		
**Age at first PAOD, N (%)**			
67–69	5823 (12.5)		
70–74	11,421 (24.4)		
75–79	11,852 (25.3)		
80–84	10,018 (21.4)		
≥ 85	7668 (16.4)		
Men, N (%)	25,043 (53.5)		
**Year of first PAOD, N (%)**			
2006	4121 (8.8)		
2007	4339 (9.3)		
2008	4651 (9.9)		
2009	4723 (10.1)		
2010	4809 (10.3)		
2011	5274 (11.3)		
2012	5357 (11.5)		
2013	5273 (11.3)		
2014	5481 (11.7)		
2015	2754 (5.9)		
**Comorbid conditions, N (%)**			
DM	27,252 (58.3)		25,234 (53.9)
Hypertension	35,795 (76.5)		32,817 (70.2)
Hyperlipidemia	12,073 (25.8)		11,413 (24.4)
**CKD severity**			
Early-stage CKD	34,476 (73.7)		37,809 (80.8)
Advanced CKD (pre-ESRD)	350 (0.8)		750 (1.6)
ESRD	11,956 (25.6)		8223 (17.6)
**Charlson comorbidity index, N (%)**			
≤ 3	26,338 (56.3)		25,061 (53.6)
4–5	12,445 (26.6)		13,829 (29.6)
> 5	7999 (17.1)		7892 (16.9)
Charlson comorbidity index, mean ± SD	3.4 ± 2.2		2.6 ± 2.0

*CKD* chronic kidney disease, *DM* diabetes mellitus, *ESRD* end-stage renal disease, *PAOD* peripheral arterial occlusive disease, *SD* standard deviation.

Supplementary Figures S2 and S3 illustrate the subgroup analyses of patients with DM and early-stage CKD, respectively. The results were identical to those of the main analysis for all patients. However, the ORs for influenza vaccination for the exposure windows of 1, 2, 3, and 4 months were not statistically significant and were close to unity among patients with advanced CKD/ESRD (Supplementary Fig. S4). Moreover, in sensitivity analysis excluding patients who were hospitalized for more than 30 days prior to PAOD onset, the ORs and 95% CIs (in parentheses) were 0.88 (0.80–0.98), 0.87 (0.81–0.95), 0.88 (0.82–0.94), and 0.89 (0.84–0.95) for the exposure windows of 1, 2, 3, and 4 months, respectively. The results were similar to those of the main analysis for all patients (Supplementary Fig. S5).

## Discussion

Our case-crossover study provided several critical findings regarding the potential protective effects of an influenza vaccination. First, elderly patients with CKD and who received influenza vaccinations exhibited a lower risk of incident PAOD within 4 months of vaccination, and these protective effects were consistently observed in patients with DM. Second, after study population stratification, in patients with advanced CKD or ESRD, influenza vaccination did not exhibit protective effects against PAOD. Our findings support the hypothesis that influenza vaccination reduces the PAOD risk only in elderly patients with early-stage CKD.

Patients with CKD are at a substantially higher risk of ASCVD^3,4,22–26^. Khalique et al.^22^ demonstrated that patients with an eGFR of < 60 mL/min per 1.73 m^2^ had a 4.1-fold higher risk of developing triple-vessel CAD than patients with an eGFR of > 60 mL/min per 1.73 m^2^. A meta-analysis including over 1.2 million participants revealed a linear association between lower eGFR and the risk of cardiovascular mortality^23^. Cerebrovascular accidents are also commonly observed in all stages of CKD. A meta-analysis of 21 studies showed that an eGFR of < 60 mL/min per 1.73 m^2^ was associated with a 43% higher risk of incident stroke^24^. Patients on dialysis have higher risks of CAD and stroke. Rostand et al. reported that 73% of patients on hemodialysis had developed significant CAD^25^. A large study based on the NHIRD in Taiwan revealed a threefold higher risk of ischemic stroke in patients on dialysis than the general population^26^. A study revealed a cumulative increase in ASCVD risk during the transition from CKD to ESRD^3^. In addition to CAD and stroke, PAOD is highly prevalent among individuals with CKD because of the shared risk factors^16,27^. In an international collaborative meta-analysis including 0.8 million individuals, patients with mild-to-moderate CKD had a 1.5–4 times increased risk of PAOD than the general population^27^.

The prevalence of PAOD is approximately 3–10%, and it increases with age^28^. Nearly 75% of patients with PAOD are asymptomatic, resulting in poor diagnosis and late treatment^29^. Symptomatic PAOD is divided into the following stages: intermittent claudication, ischemic pain at rest, and ulcer or gangrene formation^30^. Incident PAOD is associated with stroke, CAD, and all-cause mortality^11,29,31^. The etiology of ACS or stroke is the thrombotic occlusion of culprit vessels. Similarly, the presence of symptomatic PAOD is largely due to acute thrombotic events, and collateral circulation is not promptly developed to compensate for the acute ischemic burden.

Influenza vaccination has been observed to promote the stabilization of atherosclerotic plaques and generate atheroprotective immune responses in ApoE-deficient mice^32^. In a 2013 Cochrane meta-analysis of eight randomized trials, Clar et al*.* found that in patients with cardiovascular disease, influenza vaccination may reduce cardiovascular mortality and combined cardiovascular events^33^. Three large and important randomized trials, namely IAMI, IVVE, and INVESTED trials, also provide highly relevant clinical data on the efficacy of influenza as a secondary preventive measure of severe cardiovascular disease^34–36^. Chen et al. demonstrated that in elderly patients with CKD, patients receiving influenza vaccination exhibited a lower risk of hospitalization for ACS, and they noted a trend of decreased risk with an increase in the number of vaccinations^8^. As mentioned earlier in the text, the influenza vaccine is effective for ACS or stroke prevention in elderly patients with CKD. For the same reason, we assumed that the vaccine would also be effective for the prevention of acute thrombotic events in patients with asymptomatic or undiagnosed PAOD.

Although several studies have indicated that the influenza vaccine could reduce the risk of cardiovascular events and the hospitalization rates in patients with ESRD, evidence of the effectiveness of influenza vaccination for the primary prevention of ASCVD in ESRD is lacking^37,38^. This may be attributable to the considerably lower response rates of patients with ESRD to the influenza vaccine^39^.

Data on immune responses to high-dose vaccination in patients on dialysis are limited and conflicting^40,41^. Tanzi et al. reported that the administration of an additional second dose of the influenza vaccine did not improve the humoral response^40^. An observational study revealed that the administration of the high-dose influenza vaccine may be associated with lower hospitalization rates in patients on dialysis^41^. The effectiveness of the high-dose influenza vaccine among patients with ESRD remains unclear because of the lack of randomized trials; a low overall high-dose vaccination rate has been reported in a cohort study^42^.

According to vaccination guidelines by the advisory committee on immunization practices, routine influenza vaccination is recommended for all people aged ≥ 6 months^43^. Influenza vaccination is critical for individuals who are at an increased risk of severe complications from influenza, including patients who have CKD and older patients. A consensus reached by the American Heart Association/American College of Cardiology suggests that patients with ASCVD should annually receive influenza vaccination as secondary preventive measure^44^. In our study population, influenza vaccination exerted a preventive effect against the risk of incident PAOD within 4 months after exposure. A study reported that the effectiveness of vaccination declines with time, and optimum protection is achieved within 3–4 months following vaccination^45^. The antibody response in elderly patients is considerably lower than that in young adults^18^. The effects of influenza vaccination on ASCVD were observed for 1 year or more^8,36–38^. However, the immune response generated by the influenza vaccine could not be maintained for such a long duration, particularly in elderly patients. Our observation period was 4 months, during which time we evaluated the protective effects of influenza vaccination more accurately.

To our knowledge, our study is the first large study to assess the association between influenza vaccination and the risk of incident PAOD. Inflammatory processes in the pathogenesis of atherosclerosis have been well documented, and infection is one of the most common factors affecting the inflammatory process^46^. Increasing evidence supports the effect of bacterial infections and human immunodeficiency virus, hepatitis C virus, and herpes zoster infection on PAOD development^47–49^; however, data on influenza are scant and unclear. Therefore, we used the NHIRD to identify an optimal sample size and applied a case-crossover design to minimize unmeasured confounders.

The present study has several strengths; it was a large national population-based study without dropouts that used a well-established research database. We used a case-crossover design based on within-individual comparisons, which can implicitly minimize confounding bias through adjustment for time-invariant confounders. In addition, to improve the robustness of data regarding the potential protective effects of the influenza vaccine, we performed a sensitivity analysis after excluding patients with a fragile status and limited the exposure window from 2 weeks to 4 months; the results remained consistent.

This study has several limitations. First, a PAOD diagnosis was identified based on ICD-9-CM codes, and diagnostic accuracy may be a concern. Because PAOD is clinically diagnosed, we identified PAOD patients based on the presence of three or more outpatient visits or one discharge claims record to ensure that diagnoses were reliable and valid. Second, the NHIRD does not provide detailed information on health-related factors, such as cigarette smoking, nutrition status, functional status, and family history of PAOD. These unmeasured confounders were likely avoided in this case-crossover study, except that patients were consistently 1 year older in the PAOD group than in the self-control group. As a result, the cumulative effect of aging on the risk of incident PAOD could lead to the underestimation of the protective effect of the vaccine (Supplementary Fig. S6). Accordingly, although better health condition in the controls, this difference wouldn’t change our study outcome. Third, information on the effects of DM and the CKD stage is lacking in this database. A sensitivity test was conducted to investigate the aforementioned effects (Supplementary Figs. S2–S4). The results remained consistent after stratification by DM and early-stage CKD. Fourth, because of the database limitation, advanced CKD without ESA may have been classified as early-stage CKD; nevertheless, the CKD stage was determined over a period before incident PAOD diagnosis, and approximately 85% of patients with advanced CKD stage 5 received ESA therapy in Taiwan^21,50^. The impact of misclassification of the CKD stage was expected to be minimal. We believe that the large study population drawn from the entire population of a country and the exhaustive patient enrollment criteria ensured that the results were robust. Fifth, the relationship between vaccine exposure and the risk of unnoticed or asymptomatic PAOD may exist. However, the primary objective of this study was to investigate whether the influenza vaccine provides protection and prevents disease progression or acute events of PAOD. Finally, our results pertained only to elderly Taiwanese patients; therefore, the results cannot be generalized to patients aged < 65 years or different populations. The prevalence of PAOD increases with age, and influenza vaccines are mainly received by elderly people; thus, our study results are valuable. The clinical relevance of this study must be further verified by large-scale prospective randomized control trials.

## Conclusions

In the present population-based case-crossover study, we observed that elderly patients with CKD receiving influenza vaccination had a reduced risk of incident PAOD. However, the study did not include patients whose CKD had advanced to the dialysis stage or kidney transplantation. To our knowledge, this is the first large-scale study to assess the association between influenza vaccination and the risk of PAOD. The protective effect of the influenza vaccine on patients with early-stage CKD may differ according to dosage; additional clinical trials are required to confirm these benefits.

## Supplementary information

Supplementary information.

## Data Availability

The data set used in this study was based on data from the NHIRD provided by Health and Welfare Data Science Center, Ministry of Health and Welfare, Taiwan. Researchers interested in accessing this data set can submit a formal application to the Ministry of Health and Welfare to request access (https://dep.mohw.gov.tw/DOS/cp-2516-3591-113.html). All data generated or analyzed during this study are included in this article and its supplementary information files.
