# Supplementary material for: Influenza vaccination reduces incidence of peripheral arterial occlusive disease in elderly patients with chronic kidney disease
Source: Sci Rep. 2021 Mar 1;11:4847. doi: 10.1038/s41598-021-84285-8 (PMC7921588; doi:10.1038/s41598-021-84285-8)
Supplement: Supplementary file 1 — Supplementary information. [file 41598_2021_84285_MOESM1_ESM.docx]

**Influenza vaccination reduces incidence of peripheral arterial occlusive disease in elderly patients with chronic kidney disease..**

Ping-Jen Hu, Chia-Hsien Chen, Chung-Shun Wong, Tzu-Ting Chen, Mei-Yi Wu, Li-Chin Sung^*^

***Corresponding:**

Li-Chin Sung, MD, PhD ([10204@s.tmu.edu.tw](mailto:10204@s.tmu.edu.tw))

**Supplementary Information:**

Supplementary Figure S1

Supplementary Figure S2

Supplementary Figure S3

Supplementary Figure S4

Supplementary Figure S5

Supplementary Figure S6

Supplementary Table S1


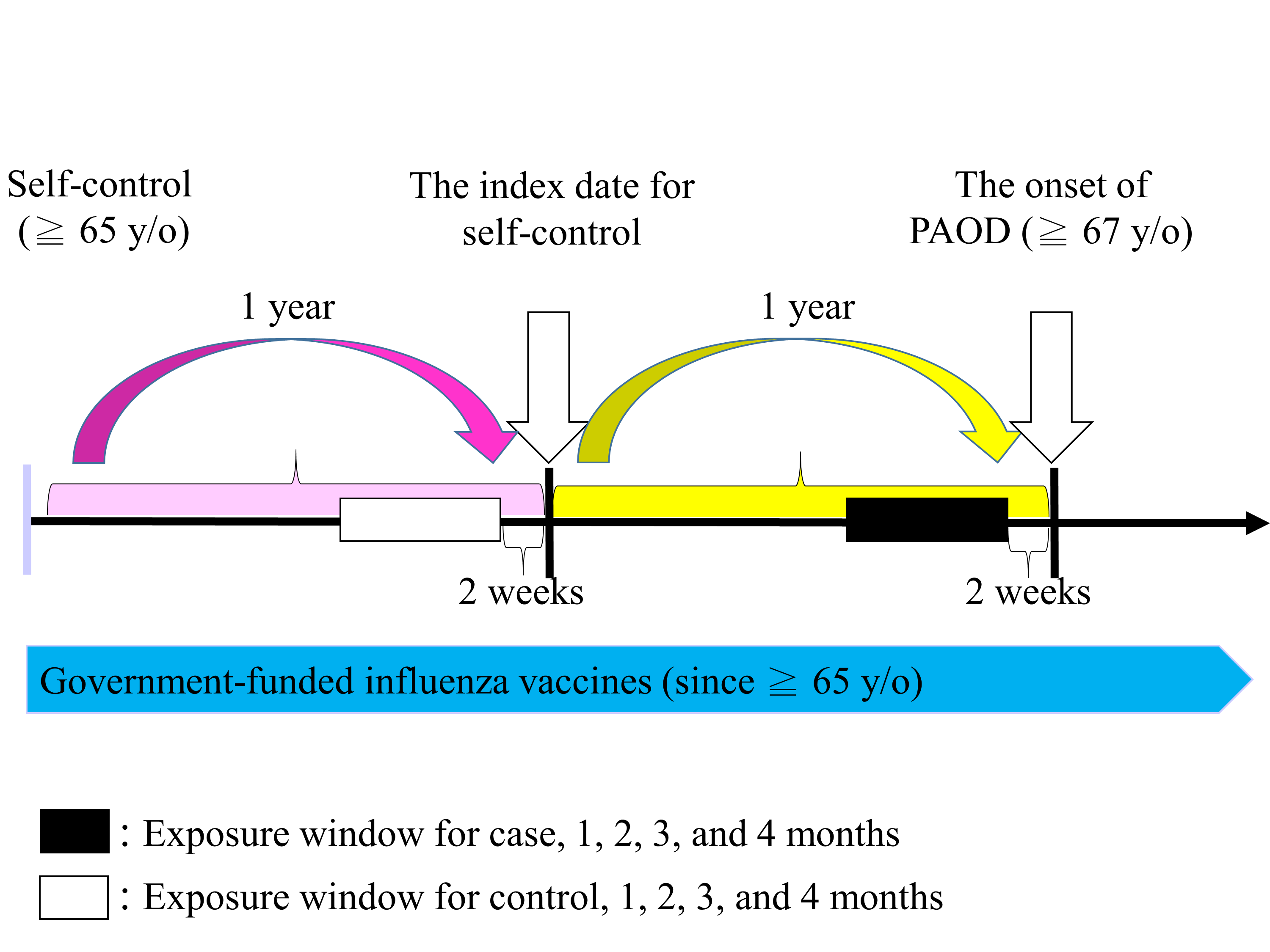


**Supplementary Figure S1.** The diagram for the relationship of incident PAOD day, index day, exposure window of influenza vaccines in both case and control groups. Government-funded influenza vaccines are free of charge for all adults older than 65 years.


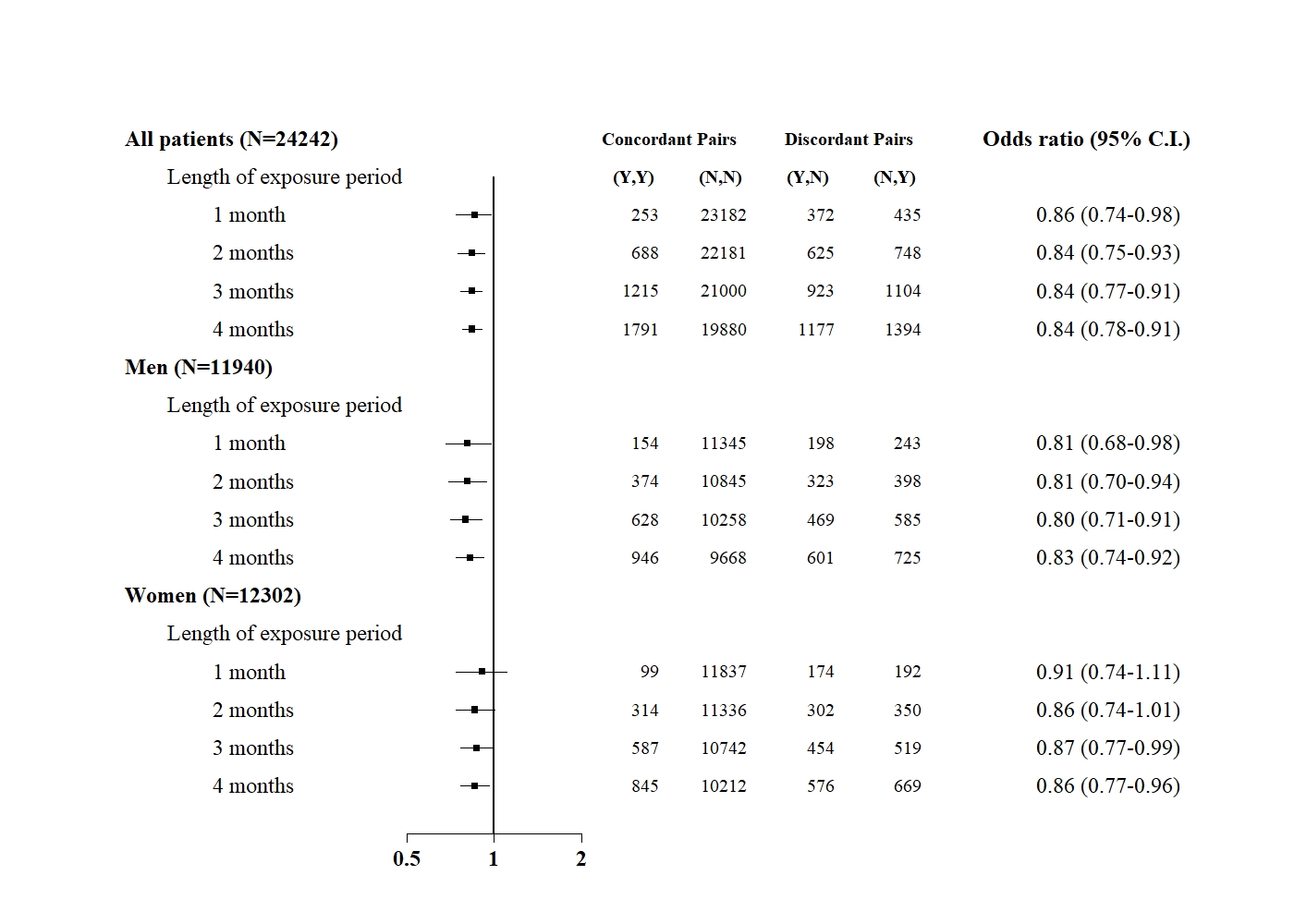


**Supplementary Figure S2.** ORs of incident PAOD after influenza vaccination in patients with CKD and DM when control was selected from 1 year before PAOD.


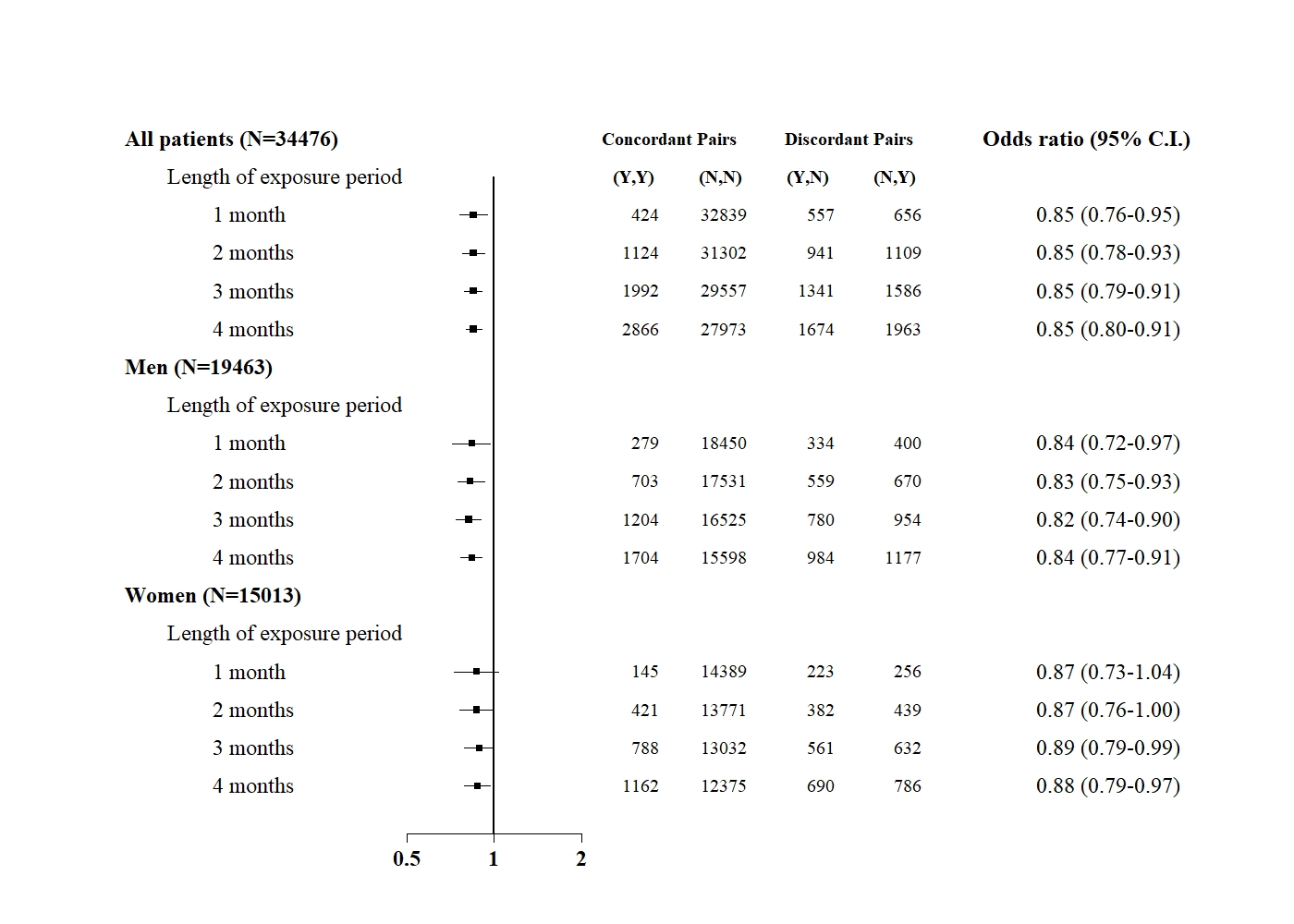


**Supplementary Figure S3.** ORs of incident PAOD after influenza vaccination in patients with early-stage CKD when control was selected from 1 year before PAOD.


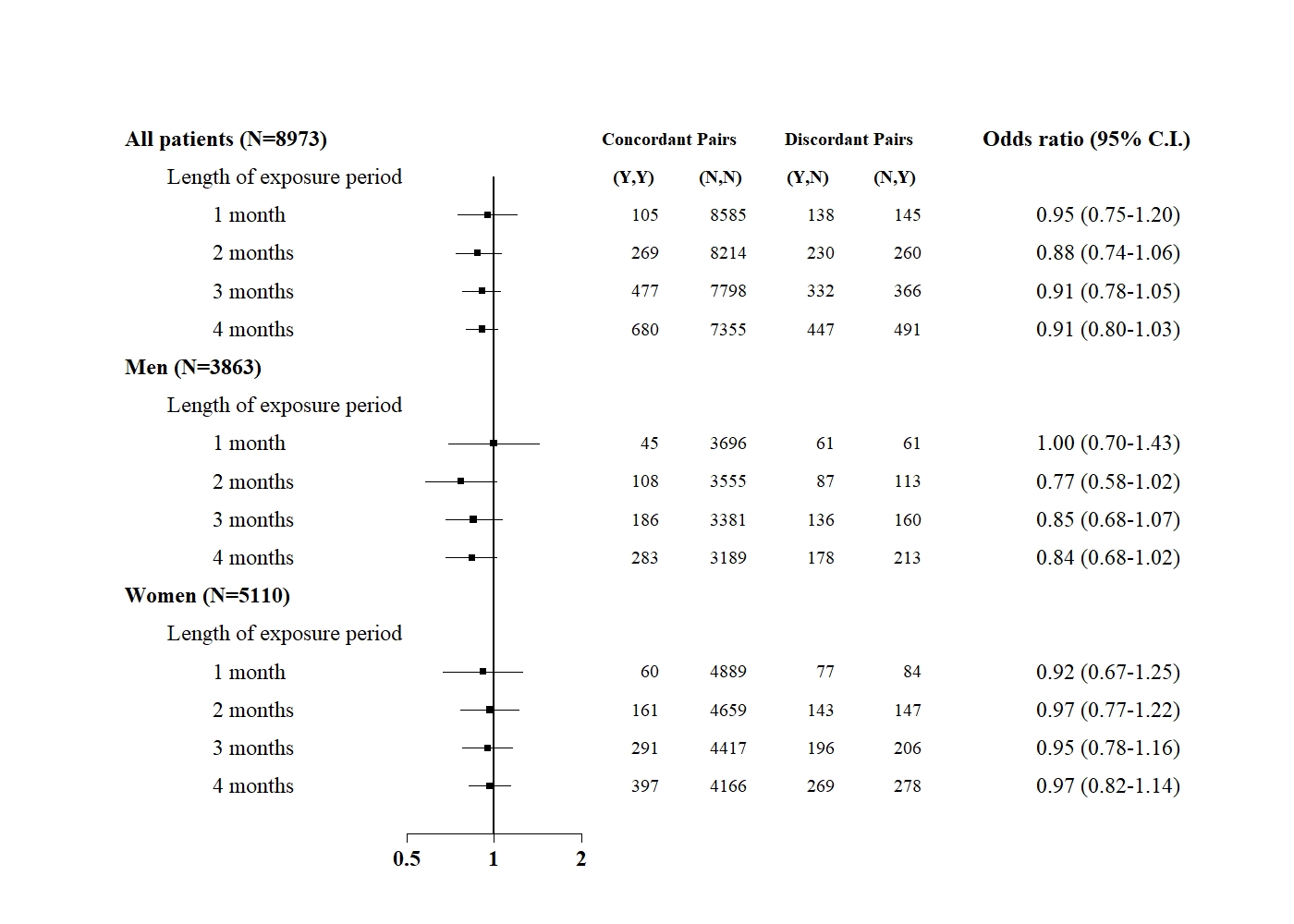


**Supplementary Figure S4.** ORs of incident PAOD after influenza vaccination in patients with advanced CKD or ESRD when control was selected from 1 year before PAOD.


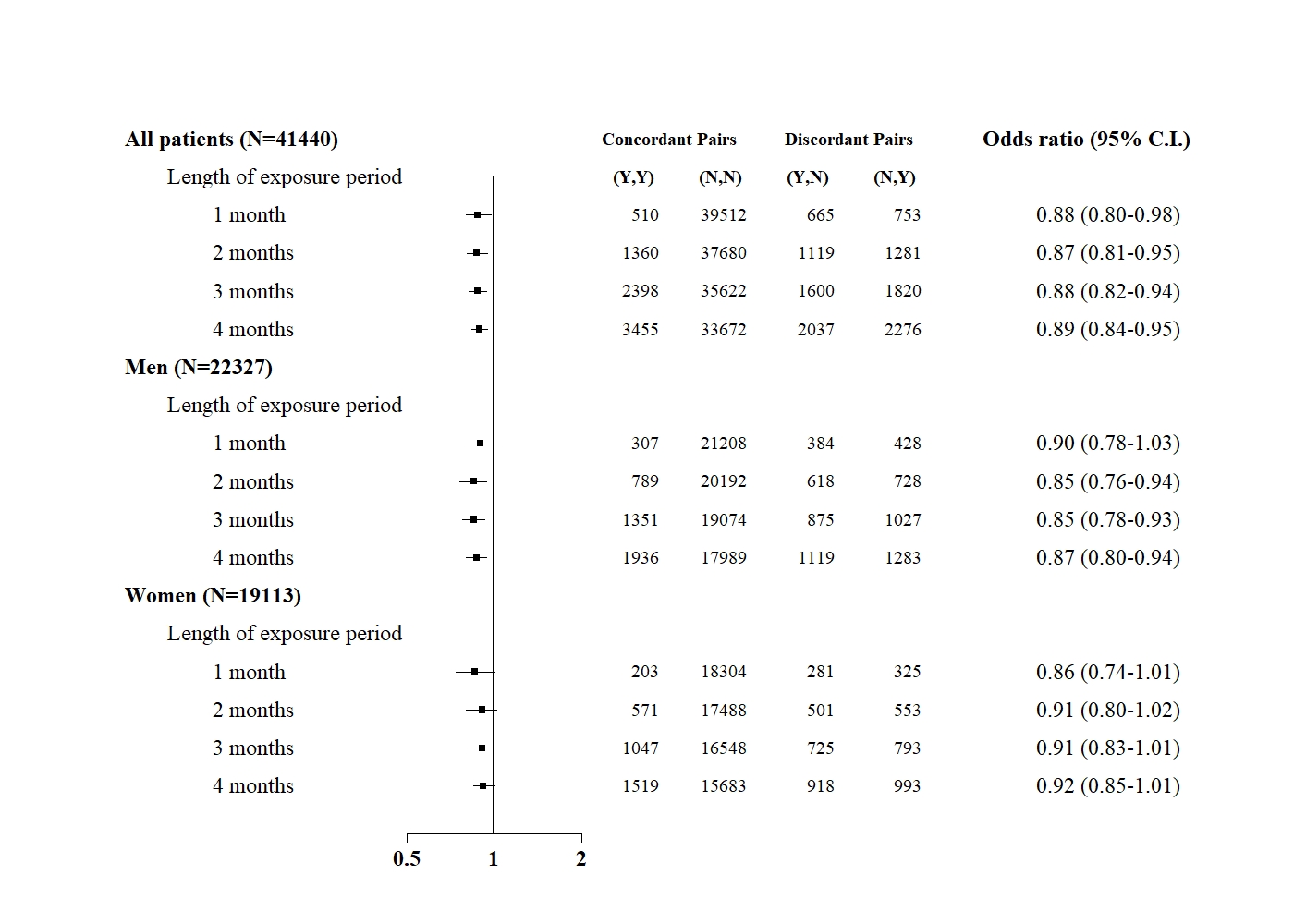


**Supplementary Figure S5.** ORs of incident PAOD after influenza vaccination for sensitivity analysis, which excluded patients who were hospitalized for more than 30 days within 1 year prior to PAOD.


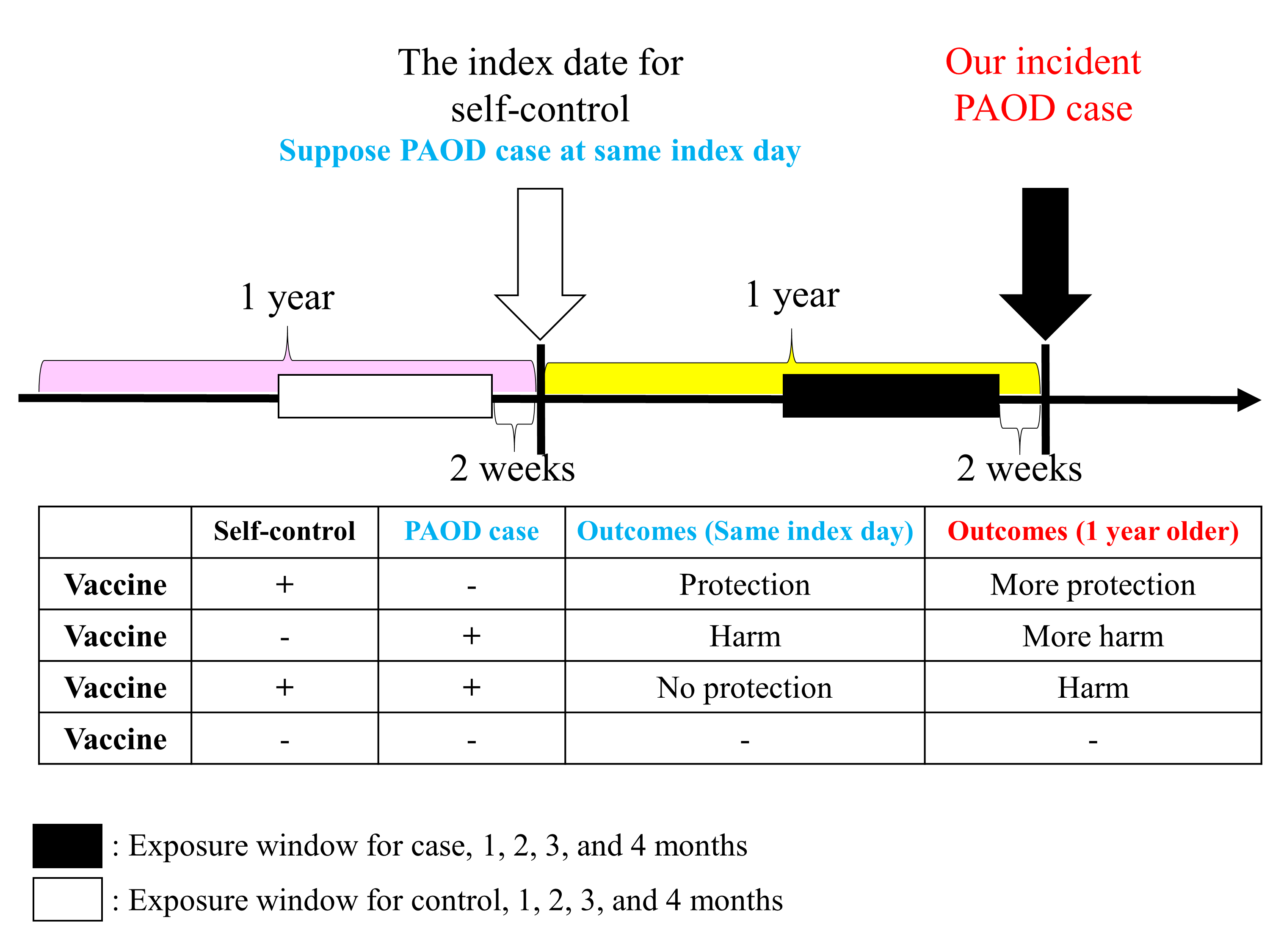


**Supplementary Figure S6.** The protective effect of influenza vaccine for the risk of incident PAOD in case group is compared with its self-control. The corresponding effects of one year aging in case group are shown in the diagram.

| **Supplementary Table S1.** Detailed information of ICD-9-CM codes |
| --- |

| **ICD-9-CM codes** | **Diseases** |
| --- | --- |
| 250 | Diabetes mellitus |
| 250.4 | Diabetes with renal manifestations |
| 274.1 | Gouty nephropathy |
| 283.11 | Hemolytic-uremic syndrome |
| 403 | Hypertensive chronic kidney disease |
| 404 | Hypertensive heart and chronic kidney disease |
| 440.1 | Atherosclerosis of renal artery |
| 440.2 | Atherosclerosis of native arteries of the extremities |
| 440.3 | Atherosclerosis of bypass graft of the extremities |
| 440.8 | Atherosclerosis of other specified arteries |
| 440.9 | Generalized and unspecified atherosclerosis |
| 443 | Other peripheral vascular disease |
| 444.22 | Arterial embolism and thrombosis of lower extremity |
| 444.8 | Embolism and thrombosis of other specified artery |
| 447.8 | Other specified disorders of arteries and arterioles |
| 447.9 | Unspecified disorders of arteries and arterioles |
| 442.1 | Aneurysm of renal artery |
| 447.3 | Hyperplasia of renal artery |
| 580 | Acute glomerulonephritis |
| 581 | Nephrotic syndrome |
| 582 | Chronic glomerulonephritis |
| 583 | Nephritis and nephropathy not specified as acute or chronic |
| 584 | Acute kidney failure |
| 585 | Chronic kidney disease |
| 586 | Renal failure, unspecified |
| 587 | Renal sclerosis, unspecified |
| 588 | Disorders resulting from impaired renal function |
| 589 | Small kidney of unknown cause |
| 642.1 | Hypertension secondary to renal disease complicating pregnancy childbirth and the puerperium |
| 646.2 | Unspecified renal disease in pregnancy without mention of hypertension |
| V04.8 | Need for prophylactic vaccination and inoculation against other viral diseases |
